# The role of epigenetics in shaping plant–mycorrhizal interactions and ecosystem resilience

**DOI:** 10.3389/ffunb.2025.1718864

**Published:** 2026-01-22

**Authors:** Aleksandra Boba, Anna Domańska, Anna Kulma, Kamila Nowosad, Kamil Kostyn

**Affiliations:** 1 Faculty of Biotechnology, University of Wroclaw, Wroclaw, Poland; 2 Department of Genetics, Plant Breeding and Seed Production, Faculty of Life Sciences and Technology, Wroclaw University of Environmental and Plant Sciences, Wroclaw, Poland

**Keywords:** defense signaling, epigenetic mechanisms, mycorrhiza, plant communication, plant–fungus interaction

## Abstract

Plants establish environmental connections through mycorrhizal symbiosis. These relationships enable them to obtain nutrients and cope with stress while simultaneously exchanging information through subterranean networks. A unified understanding of the molecular mechanisms underlying mycorrhizal interactions that drive adaptation and survival has not yet been achieved, in part because research on them stems from diverse fields of research, such as mycorrhizal ecology and plant epigenetics. This review presents recent studies demonstrating that epigenetic control serves as a central system enabling plants to adapt and maintain stable relationships with mycorrhizal fungi. We begin by describing different types of mycorrhizae. We then analyze mycorrhizal symbiosis by integrating plant and fungal genomic data with molecular evidence on DNA methylation, histone modification, chromatin remodeling, and small RNA pathways. We demonstrate that mycorrhizal symbiosis depends on changing chromatin states, which influence the regulation of the establishment, maintenance, and efficiency of symbiotic connections. They also regulate the balance between nutrient uptake and defense. They may underlie mycorrhizal stress and transgenerational “memory.” We review studies showing that RNA interference between different species enables reorganization of gene expression between plant and fungal cells. Finally, we identify key knowledge gaps and propose future research directions aimed at discovering reliable markers of mycorrhizal responses for epi-breeding and the development of climate-resilient agroecosystems.

## Introduction

Mycorrhizal symbioses form essential mutualistic bonds between plant roots and fungi. They enable plants to obtain nutrients and withstand stress, and help to maintain ecosystem balance across most terrestrial plant communities. The reciprocal exchange of resources allows fungal hyphae to extend the effective root exploration of the soil for scarce nutrients, while plants supply photosynthates to their fungal symbionts, making mycorrhizae fundamental to plant success and ecosystem functioning (Wang and Qiu, 2006; Martin and van der Heijden, 2024). At the scale of whole plant communities, extraradical mycelia create root-to-root connections that establish common mycorrhizal networks (CMNs). These networks represent a major belowground carbon pool and mediate the exchange of mineral nutrients, water and carbon compounds between conspecific and heterospecific hosts, thereby contributing to ecosystem functioning (Mahon et al., 2009; Bücking et al., 2016; Feng et al., 2019; Montesinos-Navarro et al., 2019; Song et al., 2020; Avital et al., 2022; Hawkins et al., 2023). Tracer and isotopic-labeling studies in grasslands and forests show that CMNs can redistribute carbon, nitrogen and phosphorus between plants in both directions. These exchanges range from relatively balanced reciprocity to strongly asymmetric fluxes, depending on source–sink gradients, plant identity, mycorrhizal type and fungal nutrient-allocation strategies (Grime et al., 1987; Simard et al., 1997; Simard, 2009; Philip et al., 2010; Fellbaum et al., 2014; Avital et al., 2022). CMNs mediate the balance between facilitation and competition, thereby influencing plant performance, species coexistence, and community resilience, while forming a foraging system that supports epigenetically regulated symbiotic programs (Walder et al., 2012; Montesinos-Navarro et al., 2019; Figueiredo et al., 2021; Ullah et al., 2024; Rillig et al., 2025). Mycorrhizae, together with CMNs, function as resource-exchange networks that also modulate plant defense mechanisms and signaling pathways. The presence of arbuscular mycorrhizal fungi enhances nutrient uptake, strengthens defense against insects and pathogens, and alters the release of volatile organic compounds, thereby changing how attacked plants influence their neighboring plants (Weng et al., 2022; Yu et al., 2022; Fiorilli et al., 2024). Experimental CMN systems demonstrate that defense-related cues and phytohormones can move between connected plants, triggering jasmonate- and salicylate-associated priming, and defense-gene expression, and modifying herbivore performance in receiver plants before an actual attack occurs (Babikova et al., 2013; Wu et al., 2017; Vahabi et al., 2018; Chen et al., 2019; Alaux et al., 2020; Weng et al., 2022). These “wired” interactions function as an additional form of communication, complementing volatile- and exudate-based communication, and are now understood as emergent network behaviors arising from plant–fungus associations rather than as intentional plant signals (Babikova et al., 2013; Song et al., 2014; Scott et al., 2025). Current research indicates that mycorrhizal symbioses modify both resource-distribution patterns and information networks within plant communities. This may help explain how mycorrhiza-induced priming and stress “memories” develop through synchronized chromatin and small-RNA regulation between partners, and possibly among multiple connected hosts (Figueiredo et al., 2021; Fiorilli et al., 2024; Ledford et al., 2024; Nien et al., 2024; Ullah et al., 2024; Zanetti et al., 2024; Rillig et al., 2025). Despite decades of ecological and physiological work on mycorrhizae, relatively little is known about the specific epigenetic mechanisms that regulate these symbioses. Existing evidence suggests that host-side control mechanisms, including DNA methylation, histone marks, chromatin remodelers and non-coding RNAs, operate in these symbioses. In parallel, fungal-side regulation involving chromatin architecture, diverse genomes and small-RNA signaling between kingdoms also contributes to their establishment and functioning. However, the exact control points, their temporal dynamics and mycorrhizal type-specific differences remain to be determined (Nien et al., 2024; Zanetti et al., 2024).

Epigenetics refers to the heritable, potentially reversible regulation of gene activity that occurs without changes in DNA sequence, and includes DNA methylation, histone modifications, chromatin remodeling and small RNAs. In plants, these regulatory layers tune root developmental and stress-response programs; in fungi, chromatin organization and associated regulators provide analogous control over gene expression. We adopt this mechanistic perspective to examine how epigenetic regulation could influence the initiation, maintenance and plasticity of mycorrhizal interactions (Lloyd and Lister, 2022; Zanetti et al., 2024). Despite recent conceptual and methodological advances, epigenetic aspects of mycorrhizal symbiosis are still poorly understood. Current scientific understanding treats mycorrhizal ecology and plant epigenetics as two distinct fields, failing to connect host and fungal chromatin states with small RNAs and community-level processes through common mycorrhizal networks (Lloyd and Lister, 2022; Zimmermann and Gaillard, 2023; Ledford et al., 2024; Martin and van der Heijden, 2024; Ullah et al., 2024; Zanetti et al., 2024). At the experimental level, there remains a lack of studies demonstrating how specific epigenetic changes affect symbiosis-related plant phenotypes. We also lack detailed information on epigenetic state changes that occur in both partners during the colonization and maintenance phases of symbiosis. In addition, current research is strongly biased towards a few model systems, with much less attention given to other mycorrhizal types, including EcM, ErM, OM and DSE. Moreover, there remains a shortage of time-resolved, dual-partner multi-omic studies that would establish direct links between specific epigenetic marks and symbiotic outcomes (Yildirir et al., 2022; Vigneaud et al., 2023; Fiorilli et al., 2024; Martin and van der Heijden, 2024; Somoza et al., 2024). These gaps, together with emerging opportunities from long-read and Hi-C genomics, single-cell and small-RNA methods, and integrative multi-omics, justify a focused synthesis (Wang et al., 2021; Yildirir et al., 2022; Vigneaud et al., 2023; Ledford et al., 2024; Martin and van der Heijden, 2024).

**Figure 1:**
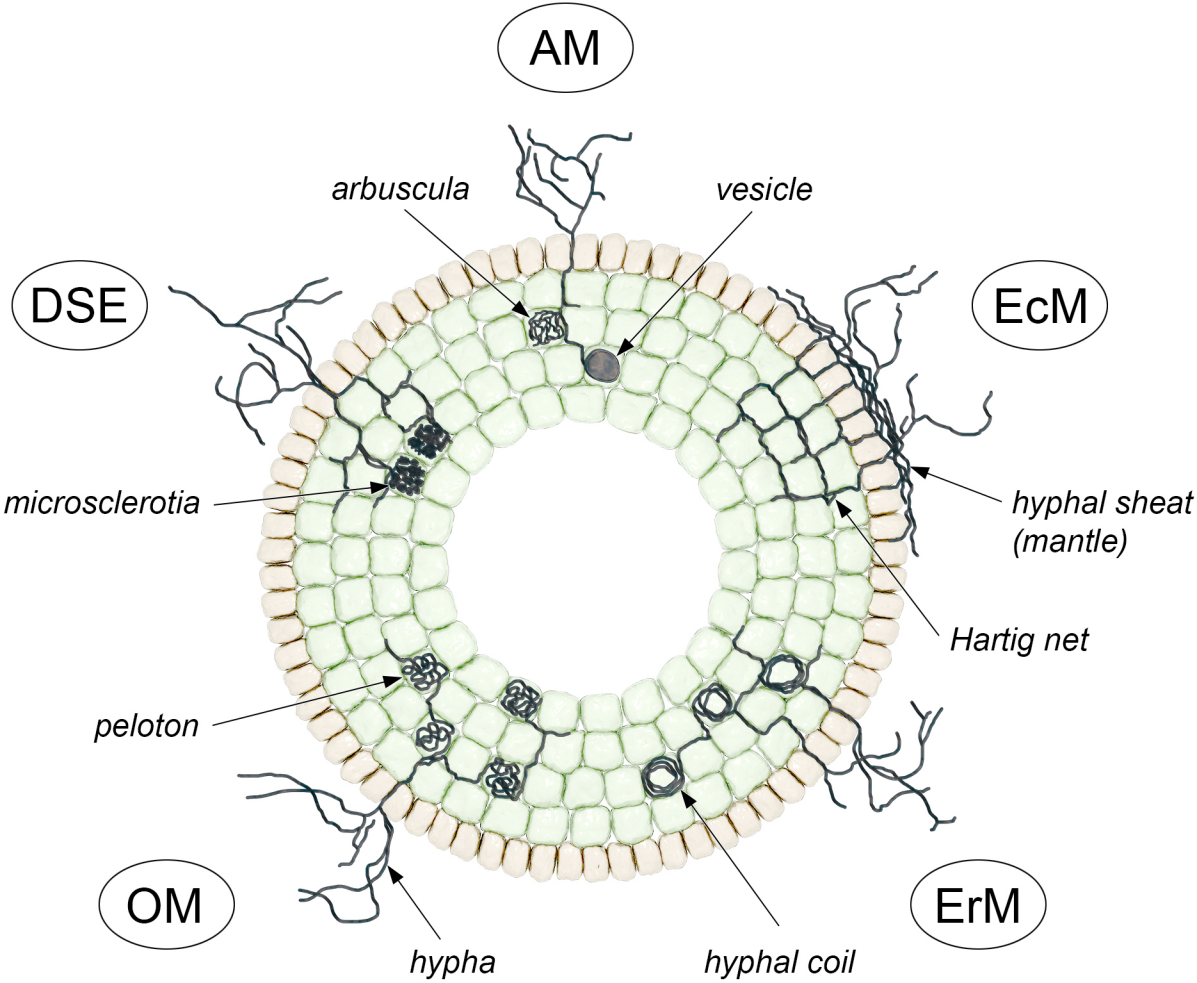
Five main mycorrhizal types which demonstrate their unique symbiosis patterns and primary exchange sites. The arbuscular mycorrhizal (AM) system creates arbuscules which establish complex networks between cortical cells for nutrient exchange and vesicles function as lipid storage compartments. The Hartig net of intercellular hyphae extends between epidermal and outer cortical cells of ectomycorrhizal (EcM) roots while the hyphal sheath (mantle) surrounds the root surface to establish the main exchange sites for carbon and minerals. The root hair cells of Ericoid mycorrhiza (ErM) contain thick hyphal coils which help them extract nutrients from acidic soils with restricted nutrient availability. The Orchid mycorrhiza (OM) system uses pelotons which are intracellular hyphal coils that periodically expand and then collapse in cortical cells to transfer carbon and nutrients to orchids that have either no chlorophyll or only partial photosynthetic ability. The root cells of Dark septate endophytes (DSE) contain melanized septate hyphae which form microsclerotia in cortical cells that serve as nutrient storage and environmental protection. The illustration presents only two root cell layers which include epidermal cells with light-brownish color and cortical cells with light-greenish appearance.

Our objectives are to synthesize current evidence on chromatin-based regulation of mycorrhiza in both plants and fungi, to evaluate indications of cross-kingdom RNA communication in these systems, and to outline research priorities, particularly time-resolved, dual-partner, multi-omic designs that link methylomes, histone marks, chromatin accessibility, and sRNA profiles with quantitative measures of colonization, resource exchange, and stress resilience. Our research primarily focuses on arbuscular, ectomycorrhizal, and other mycorrhizal associations, but we also use non-mycorrhizal fungal endophytes and beneficial root-colonizing fungi (e.g. *Epichloë* spp., SMCD 2206, *Trichoderma harzianum* T22) to demonstrate how fungal partner can modulate the host epigenome. We first provide brief context on the major mycorrhizal types (**Figure 1**) and then discuss epigenetic mechanisms in both hosts and fungi, with emphasis on key unsolved mechanistic questions.

## Mycorrhizal networks

Mycorrhizal fungi enhance plant resistance to environmental stresses, including drought and soil-borne pathogens (Bind et al., 2023). Spore germination in these fungi largely depends on the availability of carbon sources, and is stimulated by root-produced sugars, lipids, phytohormones, and fungal chitooligosaccharide signaling molecules (Gutjahr et al., 2009; Kretzschmar et al., 2012). Mycorrhizal fungi form a variety of symbiotic associations with plants, which can be classified into different types based on their morphology, physiology and host range (**Figure 1**) (Hawkins et al., 2023).

The *Glomeromycotina* subphylum (phylum *Mucoromycota*) includes arbuscular mycorrhizal (AM) fungi, such as *Rhizophagus irregularis* (formerly *Glomus intraradices*), *Claroideoglomus etunicatum* (formerly *Glomus etunicatum*), and *Claroideoglomus claroideum* (formerly *Glomus claroideum*), which form the most widespread type of mycorrhiza. These fungi establish CMNs that connect roots of both conspecific and heterospecific plant species, thereby enabling resource exchange and the transfer of defense-related signals against pathogens and herbivores (Yu et al., 2022). Fungal hyphae are attracted to the root by host-secreted strigolactones and enter through a pre-penetration apparatus, leading to the formation of arbuscules and vesicles during symbiosis. Strigolactones induce fungal hyphal branching, spore germination and effector gene expression via a Ca²^+^-mediated LysM-type receptor system, as demonstrated in the *Medicago truncatula–R. irregularis* and *Gigaspora margarita* mycorrhizal interactions (Genre et al., 2013; Mitra et al., 2021). AM fungi supply their plant partners with mineral nutrients (especially phosphate and nitrogen, in the form of ammonium and nitrate) and water, while receiving plant-derived carbohydrates (mainly hexoses) and lipids in return. Hosts commonly allocate approximately 4–20% of the carbon fixed in photosynthesis to their AM partners (Parniske, 2008; Mathieu et al., 2018; Thirkell et al., 2020).

Ectomycorrhiza (EcM) are symbioses that appeared approximately 130 million years ago from multiple fungal lineages within the *Basidiomycota* and *Ascomycota*, and now involve around 6,000 fungal species. They now form interactions with approximately 2% of plant species and primarily include the Pinaceae, Betulaceae and Dipterocarpaceae woody tree families. These symbioses function as the primary mycorrhizal network underlying forest formation in temperate and boreal regions (Lehmann et al., 2017). EcM fungi develop a mantle sheath structure around root tips while constructing a Hartig network structure which enables carbon-nutrient exchange. The extensive extraradical mycelial network, together with rhizomorphs, enables these fungi to connect plants that are spatially separated (Jörgensen et al., 2023). EcM fungi enhance nutrient uptake by their host plants and can also contribute to the remediation of contaminated soils (e.g., *Amanita muscaria* and *Pisolitus albus* when colonizing *Acacia* sp*irorbis* and *Eucalyptus globulus*) (Policelli et al., 2020). EcM fungi also emit volatile compounds, such as 1-octen-3-ol, which modulate plant growth and immunity in a concentration-dependent manner. The mutualistic communication between partners becomes visible through fungal secretion of mycorrhiza-induced small secreted proteins (MiSSPs) (e.g. from *Laccaria bicolor*), which enter plant cells to regulate defense responses and cell-wall-related gene expression (Deveau et al., 2012; Hu et al., 2022).

The fungal association known as Ericoid mycorrhiza (ErM) occurs between *Ascomycota* or *Basidiomycota* fungi and members of the Ericaceae, including blueberries, cranberries and rhododendrons (Vohník, 2020). The fungi colonize hair roots through a network of loose hyphae that produce dense intracellular “coils” which enter plant cortical cells to enable nutrient transfer. Unlike other mycorrhizal types, some ErM fungi (*Hyaloscypha* spp.) can also colonize flowers, stems, and leaves of *Vaccinium myrtillus* (Wei et al., 2022). The host plants benefit from enhanced nitrogen and phosphorus uptake provided by ErM fungi, which enable them to succeed in the poor nutrient conditions found in heathlands and similar environments (Vohník, 2020; Hu et al., 2022).

The symbiotic relationship between orchids and fungi in orchid mycorrhiza (OM) allows orchids to obtain nutrients and carbon from fungi during germination and early development, when they are fully mycoheterotrophic (Zahn et al., 2023). Some green orchids maintain mixotrophic behavior throughout their adult stage, combining photosynthesis with carbon gaining from fungi (Hossain, 2022). The partnership between these organisms improves soil nutrient acquisition and involves chemical signals that attract fungi while ensure physiological compatibility, despite the broad range of fungal associations (Sathiyadash et al., 2020).

Dark septate endophytes (DSE) are melanin-rich *Ascomycete* fungi that inhabit plant roots throughout different ecosystems including deserts, forests and grasslands (Berthelot et al., 2019). The host species determines the interaction type of DSEs, which can range from mutualistic to parasitic (Liu et al., 2022; Malicka et al., 2022). The fungi help plants absorb nutrients while making them more resistant to stresses, such as drought, salinity, heavy metals, and pathogens (Santos et al., 2021).

The various mycorrhizal associations perform essential functions by improving nutrient uptake, enabling plants to thrive in specific environments, and influencing plant health and ecosystem structure and functioning (Hawkins et al., 2023). Some plants exhibit dual-mycorrhizal status, as they can form associations with both AM and EcM fungi. Examples of dual-mycorrhizal hosts include *Populus alba* and *Populus trichocarpa*, as well as *E. globulus* and *Eucalyptus urophylla*; dual mycorrhization is also well documented in *Salix repens.* In these hosts, the relative dominance of AM vs. EcM can shift with ontogeny and environment conditions (Teste et al., 2020; Frymark-Szymkowiak et al., 2024; Nash et al., 2025). The presence of Hartig’s net and penetrating fungal hyphae in host plant roots can be observed under a microscope to confirm this dual status (Teste et al., 2020). The morphology of plant roots and associated fungal hyphae varies with the host plant’s compatibility with AM or EcM fungi. The roots of plants that associate with AM fungi become thicker but EcM fungi lead to increased mycorrhizal hyphae production (Chen et al., 2016).

## Epigenetic mechanisms regulating plant–mycorrhizal symbiosis

The establishment and maintenance of plant–mycorrhizal mutualistic relationships depend on a combination of molecular mechanisms, including epigenetic processes that regulate gene expression through complex regulatory networks. Both plants and fungi use DNA methylation, histone modifications, alternative splicing, and small RNA-mediated regulation to adapt to the demands of symbiosis. The complex interaction among these processes enables nutrient exchange and enhances stress resilience, while also controlling plant development and immune responses. This demonstrates how strongly epigenetics affects plant-fungal interactions (Mierziak and Wojtasik, 2024).

### DNA methylation changes in plant and fungal partners

#### Plant DNA methylation during mycorrhizal colonization

DNA methylation is one of the well-understood mechanisms by which plants respond to fungal colonization. Dugassa et al. (1996) discovered that flax roots exhibit decreased DNA methylation during *R. irregularis* colonization in affected root areas. Mycorrhizal systems show time-dependent DNA-methylation patterns that mirror fungal development and colonization capacity; this temporal signature is consistent with observations in *Geranium robertianum*, where colonization by the AMF *Funneliformis mosseae* triggered tissue-dependent shifts in host DNA methylation. The methylation levels in older leaves increased while root methylation decreased throughout plant aging (Varga and Soulsbury, 2019). The adaptive function of DNA methylation becomes evident through its differential regulation because it helps plants manage resource distribution between growth and defense activities during fungal colonization (Qiao et al., 2025). The expression of DNA methylation and demethylation genes shows its most significant changes during the initial step of arbuscular mycorrhizal symbiosis by *F. mosseae* in *M. truncatula* roots and then stabilizes at later stages (Hohnjec et al., 2005; Hogekamp and Küster, 2013; Volpe et al., 2023). Several important epigenetic regulators show expression changes at 10 days post-inoculation. The expression of MtDME, encoding a DNA demethylase, decreases substantially, which indicates a time-dependent reduction in demethylation activities that could maintain defense-related or stress-response gene-silencing loci. This pattern is consistent with the role of active DNA demethylases in de-repressing defense genes and with DEMETER-family functions reported in *Medicago* symbioses (Zanetti et al., 2024). DNA methylase genes responsible for maintenance of methylation, such as MtCMT2 and MtCMT3 show initial downregulation at 10 dpi before returning to baseline or slightly elevated expression levels. This indicates that methylation maintenance is temporarily relaxed to enable chromatin remodeling during symbiosis establishment before stabilization in mature root tissues (Volpe et al., 2023). Together, these findings indicate that AMS initiation involves dynamic epigenetic reprogramming through methylation-gene expression changes that occur at specific stages. The temporal expression patterns of methyltransferases and demethylases help to establish a precise chromatin environment that enables the transcriptional flexibility required for successful establishment and maintenance of fungal symbiosis (Volpe et al., 2023; Fiorilli et al., 2024; Zanetti et al., 2024).

The MSAP (Methylation Sensitive Amplification Polymorphism) technique showed that *P. alba* clone AL35 undergoes extensive DNA demethylation in leaves when mycorrhization occurs especially under heavy metal stress conditions (Cu and Zn). The observed changes became apparent only after six months of observation. The epigenetic modifications revealed gene loci that encode pseudouridine synthase and pentatricopeptide repeat (PPR) proteins involved in RNA processing, galacturonosyltransferase for cell wall metabolism and NBS-LRR proteins associated with stress resistance. The genes showed expression when AMF was present alone which indicates their adaptive nature (Cicatelli et al., 2014).

#### Transgenerational and stress-related host DNA methylation in mycorrhizal and endophytic systems

DNA methylation changes that are heritable create additional complexity in plant–fungal interactions. The AM fungal status of paternal *Geranium sylvaticum* plants determines seed methylation patterns, which affect seed viability and development. Seeds produced by hermaphroditic parents show increased DNA methylation compared to seeds from female parents, indicating that fungal-induced epigenetic changes in one generation affect the next generation. These findings indicate that AM fungi could influence plant reproduction and offspring fitness through epigenetic modifications, but the specific genes and pathways need further investigation (Varga and Soulsbury, 2017). Research on non-mycorrhizal fungal endophytes and other beneficial root-colonizing fungi provides useful reference point to study how these fungal symbionts affect plant DNA methylation patterns under stressful conditions when compared to mycorrhizal systems. DNA methylation changes caused by fungal symbionts can also be directly connected to environmental stress responses. The fungal endophyte *Epichloë* sp. LpTG-3 strain AR37 induces persistent hypomethylation in *Lolium perenne* (perennial ryegrass), which affected CHH contexts and intragenic regions across multiple plant generations. Endophyte-positive plants developed distinctive methylation patterns under drought stress which helped them become more resilient to water deficit. The observed patterns indicate that fungal endophytes help plants adapt to abiotic stress through epigenetic landscape reprogramming which stabilizes stress related gene expression pathways (Forte et al., 2023). *Triticum turgidum* (wheat) seedlings treated with the fungal endophyte SMCD 2206 which confer drought tolerance, show improved drought resistance with higher seed germination rates and reduced stress damage compared to non-inoculated seedlings. The methylation status of endophyte-free seedlings under drought conditions showed a significant decrease in semi-methylated sites when compared to other treatment groups. In contrast, the methylation patterns of inoculated seedlings under drought stress matched those of non-stressed plants at more than 95% of examined loci which suggests that SMCD 2206 maintains genomic stability by controlling transposable elements (TEs) mobility and modulating other epigenetic factors that affect gene expression and genome integrity. Thus, the agricultural potential of SMCD 2206 lies in its ability to maintain a non-stressed epigenetic state while offering drought resistance (Hubbard et al., 2014). Beyond mycorrhizal systems, beneficial root-colonizing fungi provide further insight into the epigenetic control of plant–fungus interactions. The epigenetic modifications in tomato roots that interact with *Trichoderma harzianum* T22 follow a time-dependent pattern which corresponds to the progression of colonization and defense priming. The activation of systemic acquired resistance (SAR) genes occurs during early interaction stages when DNA hypomethylation becomes evident. The plant immune system receives an early demethylation signal which helps it prevent uncontrolled fungal growth. After the initial interaction with the beneficial fungus, the plant progressively re-balances hormone signaling (particularly SA–JA–ET crosstalk), redox status and defense-related gene expression, while re-activating growth- and nutrient-related transcriptional programs. or suppresses specific defense responses through hypermethylation during the later stages (48–72 hpi) to maintain symbiotic balance (De Palma et al., 2019).

Similar host-side changes of DNA methylation have been observed in EcM interaction between poplar and *L. bicolor* as the fungal partner. Scientists used transgenic poplar lines with modified DNA methylation states, through DDM1 and DML gene overexpression or RNA interference, to study the effects of hypo- and hypermethylation. The modifications affected both the development of ectomycorrhizal structures and the extent of fungal penetration into plant tissues (Hartig net depth). The formation of ectomycorrhizae and fungal penetration was reduced in poplar transgenic lines with decreased methylation levels. The interaction between poplar and *L. bicolor* resulted in modified DNA methylation patterns that showed how host and fungus epigenetic changes depend on each other (Vigneaud et al., 2023). These findings show that fungal partners (both mycorrhizal and symbiotic) can trigger local, systemic, and even heritable changes in host methylomes.

#### Fungal DNA methylation and genome compartmentalization in arbuscular mycorrhizal fungi

DNA methylation differences extend beyond cytosine because scientists now focus on studying adenine methylation. Research on N6-methyladenine (6mA) in the model AMF *R. irregularis* has shown that variation in 6mA levels may affect symbiotic outcomes. The two genetically identical clones C2 and C5 showed minimal variations in 6mA methylation density which affected the expression of symbiosis-related genes including phosphate transporter genes. The dikaryotic isolate C3 displayed methylation heterogeneity between its two haploid nuclear genotypes. The observed epigenetic variations between genetically identical clones may help explain their different effects on host growth (Chaturvedi et al., 2021). The genomic structure of AM fungi reveals their ability to adapt through epigenetic mechanisms. Recent genomic and transcriptomic data show that heterokaryotic isolates of *R. irregularis* exhibit different epigenetic profiles. The symbiotic genes which include effectors and secreted proteins are mainly found in heterochromatic regions known as B compartments. This region contains repetitive elements, show higher epigenetic variability and have their expression tightly controlled. The host signals during plant colonization trigger changes in the epigenetic state of this compartment which enables the fungus to activate essential symbiotic genes. The compartmentalized regulation system demonstrates how fungal genomes can adjust to different host environments through their flexible genetic organization (Yildirir et al., 2022; Sperschneider et al., 2023).

The *R. irregularis* genome contains 23–43% repetitive sequences along with numerous transcriptionally active TEs that emerge during spore development. The two main epigenetic control mechanisms that regulate these elements include cytosine DNA methylation and small RNA-mediated silencing. The genome contains two distinct gene categories: core (conserved/accessory) and noncore (often unannotated or orphan) genes, which occupy different spatial regions. Noncore genes often occur in repeat-rich regions that have high methylation levels. The second mechanism which is RNA interference plays also a crucial role in TE regulation. The *R. irregularis* genome contains at least 30 Argonaute (AGO) protein paralogs together with Dicer (DCL) and the sRNA methyltransferase HEN1 which are part of the RNAi machinery (Hung and Slotkin, 2021). Proteomic and small RNA profiling of spore development shows that these components were actively expressed. The majority of sRNAs derive from TEs, especially young LINE and Gypsy retrotransposons, and from unannotated regions that are often found near TEs, indicating RNAi as the primary defense mechanism against transposon activity. The absence of sexual reproduction in *R. irregularis* makes TE activity the primary source of genetic variation and adaptation. The expansion of orphan and accessory genes that participate in host interaction is supported by TEs dynamics, together with horizontal gene transfer and cryptic recombination (Dallaire et al., 2021). Recent genomic studies of AMF confirm that nuclear dynamics together with TEs and epigenetic plasticity generate most of the genetic diversity in AMF species that reproduce asexually. The wide host range and environmental adaptability of AM fungi is supported by genome and epigenome organization, which modulate their ability to respond to changing environmental conditions through epigenetic regulation (Lanfranco and Bonfante, 2023).

### Chromatin remodeling and histone modifications in plant and fungal partners

Histone modifications represent another crucial epigenetic mechanism by which fungi manipulate plant gene expression during colonization. However, epigenetic modifications of histones are better known from pathogenic interactions between plants and fungi and are described in detail in the work of Lai et al. (2022).

#### Plant chromatin remodeling and histone marks

Evidence for a role of histone acetylation in mycorrhizal regulation comes from *Citrus sinensis*. Shu et al. (2021) found 14 histone acetyltransferases (CsHATs) and 11 histone deacetylases (CsHDACs) in sweet orange plants and demonstrated that arbuscular mycorrhizal colonization with *F. mosseae* changes their expression levels through induction of HAT genes (CsHAT1, 2, 6, 7, 9, 11, 12 and 14) and the repression of HDAC genes (CsHDAC1, 2, 5, 6, 8 and 10) in mycorrhizal roots. Plant HATs and HDACs show mycorrhiza-responsive expression through stress- and signaling-related cis-elements in their promoters which indicates their role in chromatin-based transcriptional reprogramming during AMS in perennial woody hosts (Shu et al., 2021). Research on *M. truncatula* plants shows that plant histone deacetylases control the formation of symbiotic structures which develop inside plant cells. The PhD research of Li (2017) demonstrated that MtHDT genes are expressed in the nodule primordium and infection zone. In the *Medicago*–*R.irregularis* arbuscular mycorrhizal symbiosis, MtHDT2 is specifically induced in arbuscule-containing cortical cells. RNAi knock-down of MtHDTs leads to nodule development defects and rhizobial infection problems through decreased MtHMGR1 expression which interacts with MtDMI2. Knockout of MtHDT2 reduces arbuscule number and increases their collapse in infected roots without affecting overall *R. irregularis* colonization. HDTs regulate chromatin states that direct the formation of symbiotic structures in nodules and arbuscular mycorrhizal roots (Li, 2017). Transcriptomic and cell-biological studies also point to histone gene regulation as part of the host nuclear response to AM fungi. Siciliano et al. (2007) used the PPA as a marker to identify specific genes which express differently in *M. truncatula* epidermal cells beneath hyphopodia formed by *G. margarita*. The histone H2B1 gene is specifically activated in root sections where appressorium structures and PPA assembly occur, which indicates cell cycle-dependent transcriptional activation during the initial stages of interface development. Russo and Genre (2021) expanded this work by showing that, in *M. truncatula*, arbuscular mycorrhizal colonization triggers ectopic cell divisions and local endoreduplication in inner cortical cells. They also observed increased expression of the histone gene MtHist-H4 and endocycle regulators such as MtAPC/C subunit 2, MtCCS52A and DNA topoisomerase VI subunits. The research confirms that AM development needs specific root cortical cell layers to restart their cell cycle and up regulate histone gene expression. This, in turn, generates an active nuclear environment for chromatin-based control of symbiosis-related genes (Russo and Genre, 2021).

#### Fungal chromatin states and effectors targeting host histones

*R. irregularis* produces the effector RiNLE1 which serves as a striking example of mycorrhizal fungus histone modification. The effector protein RiNLE1 enters plant nuclei and blocks histone H2B mono-ubiquitination. The overexpression of RiNLE1 results in enhanced hyphal colonization, without affecting arbuscule abundance. This finding confirms the role of this effector in promoting fungal colonization and strengthening mutualistic relationships. The study of RiNLE1 inducible expression revealed its ability to suppress host defense and stress-related genes which provides insights into its function in AM colonization host-pathogen interactions. These histone-targeting mechanisms demonstrate the advanced methods fungi use to manipulate host epigenetic regulation for their own advantage (Wang et al., 2021). The symbiotic relationship between *E. festucae* fungal endophytes and *L. perenne* grass is based on the fungal biosynthesis of bioprotective alkaloids which protect the host from various biotic and abiotic stresses in exchange for primary metabolites from the plant. Trimethylation of histone H3 at lysines 9 and 27 (H3K9me3 and H3K27me3) is reduced in the fungus in response to host signals, which enables specific secondary metabolism during symbiosis. This chromatin-based regulatory system plays an essential role in both metabolite production and in the development and maintenance of mutualistic relationships with the grass host (Chujo and Scott, 2014).

### RNA interference and cross-kingdom small RNAs in plant–fungus interactions

#### Fungal small RNAs and cross-kingdom RNAi

Small RNA molecules function as essential mediators of cross-kingdom communication between fungi and plants. Research on *R. irregularis* has shown that the fungus contains an active RNAi pathway which produces sRNAs that can target plant mRNAs (approximately 237 plant genes were predicted as putative target in *M. truncatula*). These fungal sRNAs control host metabolic pathways and defense responses by silencing specific plant genes during colonization. sRNA sequences from *R. irregularis* have been proven to target *M. truncatula* transcripts including those related to immune responses. Several predicted targets of Rir-sRNAs are involved in key processes during AMS and plant responses to stress. An example is Specific Tissue Protein 6, which is a target of Rir-sRNA (3121–59), involved in the dynamic regulation throughout the various stages of symbiosis. The Responsive to Dehydration 22 (RD22) gene, linked to ABA-dependent signaling for abiotic stress tolerance, is downregulated in arbusculated cells, likely due to targeting by three specific Rir-sRNAs 773–218, 10,035–21, 10,035–21. The leucine-rich repeat (NBS-LRR) disease resistance gene that resembles the rice OsRGA3 blast resistance gene is suppressed by Rir-sRNA 7710–27 to enable fungal colonization through reduced host defense mechanisms (Silvestri et al., 2019). This RNA-based mechanism enables the fungus to disable host defense for successful symbiotic establishment. Ectomycorrhizal fungi also use small RNAs to modify host root functions through cross-kingdom RNA transfer. The fungus *Pisolithus microcarpus* contains 11 miRNAs including Pmic_miR-8 which accumulates in *Eucalyptus grandis* root cells upon colonization. *In situ* hybridization experiments demonstrated that Pmic_miR-8 originates from fungus and shows distribution patterns similar to AGO protein which indicates its integration into the host RNAi machinery. Suppression of Pmic_miR-8 results in enhanced root regrowth after mantle formation, together with decreased formation of stable mycorrhizal root tip, which indicates unstable colonization. These results indicate that Pmic_miR-8 functions to sustain ECM colonization by suppressing host immune components including CC-NLR-type resistance genes (e.g., RGA4, RGA2) which were identified through RNAseq analysis (Wong-Bajracharya et al., 2022).

#### Plant small RNAs in mycorrhizal symbioses

Plant microRNAs function to control fungal colonization at levels that prevent excessive fungal growth. The transcription factor NSP2 in *M. truncatula* is targeted by miR171h which functions as a key regulator of mycorrhizal symbiosis. The expression of NSP2 remains suppressed by elevated miR171h levels which restricts fungal growth in root elongation zones, whereas NSP2-resistant plants show increased fungal colonization. This regulatory system maintains a proper symbiotic equilibrium which stops fungal overgrowth in plant tissues (Lauressergues et al., 2012). The regulatory complexity of mycorrhizal interactions becomes evident through the study of miR396 and other miRNAs. The root meristem activity of *M. truncatula* is controlled by miR396 through its repression of growth-regulating factors that drive cell proliferation. The efficiency of *R. irregularis* mycorrhizal colonization decreases when miR396 is overexpressed whereas the colonization rate increases when miR396 is inactivated. Importantly arbuscule formation isn’t affected, which indicates that miR396 functions differently in mycorrhization compared to nodulation in *M. truncatula* (Bazin et al., 2013a). The miRNA networks of *Poncirus trifoliata* were studied to show their role in AM symbiosis with *R. irregularis* DAOM197198. The established miRNA families miR399 and miR171 are known to be important in AM symbiosis, but other miRNA families such as miR156, miR160, miR166, miR164, miR172, miR319, miR396, miR395, miR408, and miR827 were also found to be involved in these processes. The miRNA families miR477, miR390, and miR1446 are specific to *P. trifoliata* and are involved in AM symbiosis in citrus plants. The overexpression of miR477 reduces arbuscule abundance and affects lateral root development and AM colonization. The stable transformation of *P. trifoliata* with miR477 confirmed its role in regulating AM development. miRNA-mRNA network analysis revealed that PtSUT2 (sucrose transporter), PtAMT2;3 (ammonium transporter), and PtEXO70I (exocytosis-related protein) are regulated by miRNAs such as miR159, miR167, and miR477. miR167 regulates nitrogen transport and hormone metabolism, while miR477 regulates arbuscule development by targeting transcription factors RAD1 (REQUIRED FOR ARBUSCULE DEVELOPMENT 1). These findings suggest a coordinated action of miRNAs and their transcription factor targets in AM symbiosis, revealing species-specific regulatory mechanisms that may be critical for citrus plants (Ji et al., 2023). Root miRNA profiles of *Nicotiana attenuata* plants change during *R. irregularis* colonization. These changes differ between plants with complete colonization and those with impaired colonization (mutant lines that have silenced calcium- and calmodulin dependent protein kinase (CCaMK) expression). The symbiosis regulatory miRNA families miR156, miR171, miR393, miR399, miR472/482 and miR172 showed differential expression. The GRAS transcription factors NSP2 and DELLA, which are crucial for AMF signaling and arbuscule development, show specific enrichment of miR171 and miR473 in fully colonized roots. The nutrient acquisition deficiency of irCCaMK plants matched with accumulation of miR399, which is linked to phosphate starvation. The AMF-inoculated roots showed increased levels of miR482 and miR8667, which target NBS-LRR-type resistance genes thus indicating their role in defense suppression and symbiosis balance. These results demonstrate that AMF-triggered miRNAs operate through complex regulatory networks to control nutrient uptake, development and immune responses in a specific manner (Pandey et al., 2018). A total of 432 miRNAs were identified in *Populus tomentosa* roots colonized by *Cenococcum geophilum*, including 156 novel sequences. Among these, 51 were differentially expressed (DE-miRNAs) in response to ECM colonization. Degradome and computational analyses confirmed hundreds of miRNA–target interactions. The upregulation of miR164 correlated with the downregulation of NAC transcription factors which matches the defense responses observed during pathogenic fungal interactions with *Magnaporthe oryzae* and *Verticillium dahlia* (Wang et al., 2018; Hu et al., 2020; Tao et al., 2023). The downregulation of miR393c occurred while its auxin-related targets showed increased expression which could indicate a release of repression on pathways that promote symbiotic colonization (Etemadi et al., 2014). The expression pattern and response mechanism of this miRNA shows different patterns in various plant–microbe interactions (Yang et al., 2013; Tao et al., 2023). The DE-miRNAs miR319, miR394, miR396, miR397 and novel miRNAs miR44 and miR80 were found to be involved in processes like lignin degradation, flavonoid metabolism, and nutrient transport processes. The functional enrichment analysis showed that DE-miRNA targets are mainly found in pathways essential for EcM symbiosis including phenylpropanoid biosynthesis, cell wall remodeling, iron/copper homeostasis. Several *P. tomentosa* miRNAs were predicted to target fungal genes which indicates cross-kingdom RNA interference. These findings demonstrate that miRNAs play a crucial role in managing plant–fungus communication and metabolic coordination and stable ectomycorrhizal symbiosis establishment (Tao et al., 2023). High-throughput sequencing analysis of *Rubus idaeus* (raspberry) tissue inoculated with DSE strain *Phialocephala fortinii* F5 revealed 361 known and 95 novel miRNAs with 34 highly conserved miRNA families including miR159, miR166, and miR396 showing high abundance (Yang et al., 2022). The identified miRNAs control essential developmental processes and stress response mechanisms. The MYB transcription factors are targets of miR159 which controls flowering, fruit development and stress tolerance (Palatnik et al., 2007; Millar et al., 2019). By contrast, miR166 controls root architecture and miR396 affects root development and mycorrhizal colonization in *M. truncatula* (Boualem et al., 2008; Bazin et al., 2013b). DE analysis across the three sample groups, which included controls, early (3 weeks) and late (5 weeks) stages of symbiosis, revealed dynamic and stage-specific miRNA regulation (Yang et al., 2022). The conserved miRNAs miR159, miR166, miR171 and miR396 target transcription factors that regulate root development and symbiotic control through MYB, HD-ZIP III, NSP2 and GRF (Li et al., 2017). The conserved miR171–NSP2 regulatory complex, known from legume–mycorrhizal systems, was also confirmed in raspberry, suggesting a shared mechanism of fungal recognition (Lauressergues et al., 2012).

## Conclusion and future perspectives

Current research shows mycorrhizal symbioses function as flexible networks that mediate not only nutrient and water exchange, but also signal communication and epigenetic regulation between plant and fungal partners. Both organisms employ epigenetic mechanisms, including DNA methylation, histone modifications, chromatin remodeling and non-coding RNAs, to exchange molecular signals that influence colonization and responses to environmental stresses (Zimmermann and Gaillard, 2023; Fiorilli et al., 2024; Martin and van der Heijden, 2024).

Mycorrhizal interactions induce changes in DNA methylation and chromatin states that allow root cells and fungal structures to reorganize for the establishment and maintenance of symbiosis. These changes are linked to hormone signaling pathways, defense mechanisms, and nutrient transport systems, and can be reinforced or relaxed depending on environmental conditions. The epigenomes of arbuscular mycorrhizal fungi and other symbiotic fungi show dynamic behavior, which helps them adapt to different soil environments, yet their genome-scale characteristics remain understudied (Fiorilli et al., 2024; Díaz et al., 2025; Karalija et al., 2025).

Small RNAs have been identified as essential regulators, which control mycorrhizal relationships between plants and fungi. The process of mycorrhization induce plants to produce miRNAs and siRNAs which target genes responsible for root development, nutrient absorption and immune response. Research studies show cross-kingdom RNA interference as a part of plant–microbe interactions, but additional quantitative data are still needed to characterize these processes in mycorrhizal ecosystems (Rabuma et al., 2022; Ledford et al., 2024; Liu et al., 2025).

Although the epigenetic field has made significant progress, several critical knowledge gaps remain unresolved. There is an urgent need to develop single-cell and spatially resolved epigenomic approaches for mycorrhizal root cells, as well as their surrounding tissues. Resent works has shown that single-cell transcriptomics, ATAC-seq (Assay for Transposase-Accessible Chromatin) and spatial multi-omics techniques enable the dissection of plant–microbe endosymbiosis regulatory states at cellular resolution. These approaches have already been used to study arbuscular mycorrhizal roots through transcriptomic analysis (Martin and van der Heijden, 2024; Serrano et al., 2024; Somoza et al., 2024; Ferreras-Garrucho et al., 2025). However, single-cell-resolution maps of DNA methylation, histone modifications, and small RNA profiles are still lacking. Developing effective methods to isolate colonized cortical cells, distinct arbuscule stages and fungal structures from root tissues is essential to characterize their epigenomic landscapes and link them to nutrient exchange, immune modulation, and fungal development. Moving beyond bulk tissue analysis toward spatially and temporally resolved measurements will greatly advance our understanding of epigenetic control mechanisms operating at specific stages and locations of symbiotic interactions.

A second major challenge is to determine which mycorrhiza-induced epigenetic marks are heritable and to what extent. Research on gynodioecious *G. sylvaticum* has demonstrated that seed DNA methylation patterns vary with parental mycorrhizal status, indicating that symbiosis can establish an epigenetic link to the next generation (Varga and Soulsbury, 2019). Studies of epigenetic stress memory and transgenerational phenotypic inheritance show that some environmentally induced epigenetic changes can persist across multiple generations, whereas many others are reset or impose fitness costs (Kovalchuk, 2023; Karalija et al., 2025; Latzel et al., 2025). It remains unclear which mycorrhiza-induced DNA modifications and chromatin rearrangements become sufficiently stable to be transmitted across generations, how long they persist, and what adaptive value they confer under different environmental conditions. Addressing these questions will require multigenerational studies that combine mycorrhizal treatments with DNA and chromatin profiling, alongside measurements of fitness and reproductive traits in natural field settings.

A third key future direction is to apply epigenetic knowledge to the development of environmentally friendly agricultural practices. Research indicates that arbuscular mycorrhizal fungi enhance crop productivity under drought, salinity, extreme temperatures, and heavy metal contamination, while optimizing nutrient use and improving disease resistance, making them well suited for sustainable low-input farming systems (Birhanu and Negussie, 2024; Chowdhary and Songachan, 2025; Kumar et al., 2025; Samanta et al., 2025). In parallel, reviews on epigenetic stress memory and crop resilience show that heritable or semi-stable epigenetic marks can underpin long-lasting defense priming and enhanced tolerance to both abiotic and biotic stresses (Karalija et al., 2025; Thingujam et al., 2025). However, the connection between these two research fields remains poorly developed. Three key questions need to be: (1) whether mycorrhizal colonization can serve as an epigenetic priming strategy for crops; (2) how stable and effective such priming is across seasons and plant generations; and (3) which specific epigenetic markers reliably indicate “mycorrhizal responsiveness” and improved crop performance under field conditions.

From an applied perspective, future work will need to:

1. Identify specific epigenetic markers that indicate beneficial mycorrhizal interactions across crop genotypes, including analyses of DNA methylation, histone modifications, and small RNA expression, to enhance nutrient uptake and stress tolerance (Wang and Bart, 2025).
2. Integrate these epigenetic markers into breeding and epibreeding programs, enabling the selection of plant genotypes that consistently benefit from mycorrhizal symbioses through genomic and epigenomic selection approaches.
3. Assess how agricultural practices, such as fertilizer application, crop rotation, and pesticide use affect plant-mycorrhizal communities and their epigenetic responses, using extensive field-based experiments that combine mycorrhizal analyses with multi-omics data integration (Chowdhary and Songachan, 2025).
4. Explore, over the longer term, targeted epigenome editing and RNA-based approaches (including cross-kingdom RNA interference) as tools to fine-tune symbiotic signaling, while carefully evaluating potential negative outcomes and regulatory constrains (Liu et al., 2025).

Epigenetic mechanisms function as essential regulators of mycorrhizal processes, governing host-fungal interactions and their effects on ecological systems and overall ecosystem functioning. Addressing these key questions will help identify mycorrhiza-induced epigenetic states that are stable across generations and capable of supporting resilient agroecosystems, from single root cells to entire plant communities. Achieving this goal will require integrating single-cell and spatial epigenomics with multi-omics analyses, multigenerational field studies, and epigenetically informed breeding and management strategies, thereby the full exploitation of mycorrhizal symbioses for sustainable agriculture and climate-resilient ecosystem development.

## Figures and Tables

**Figure 2:**
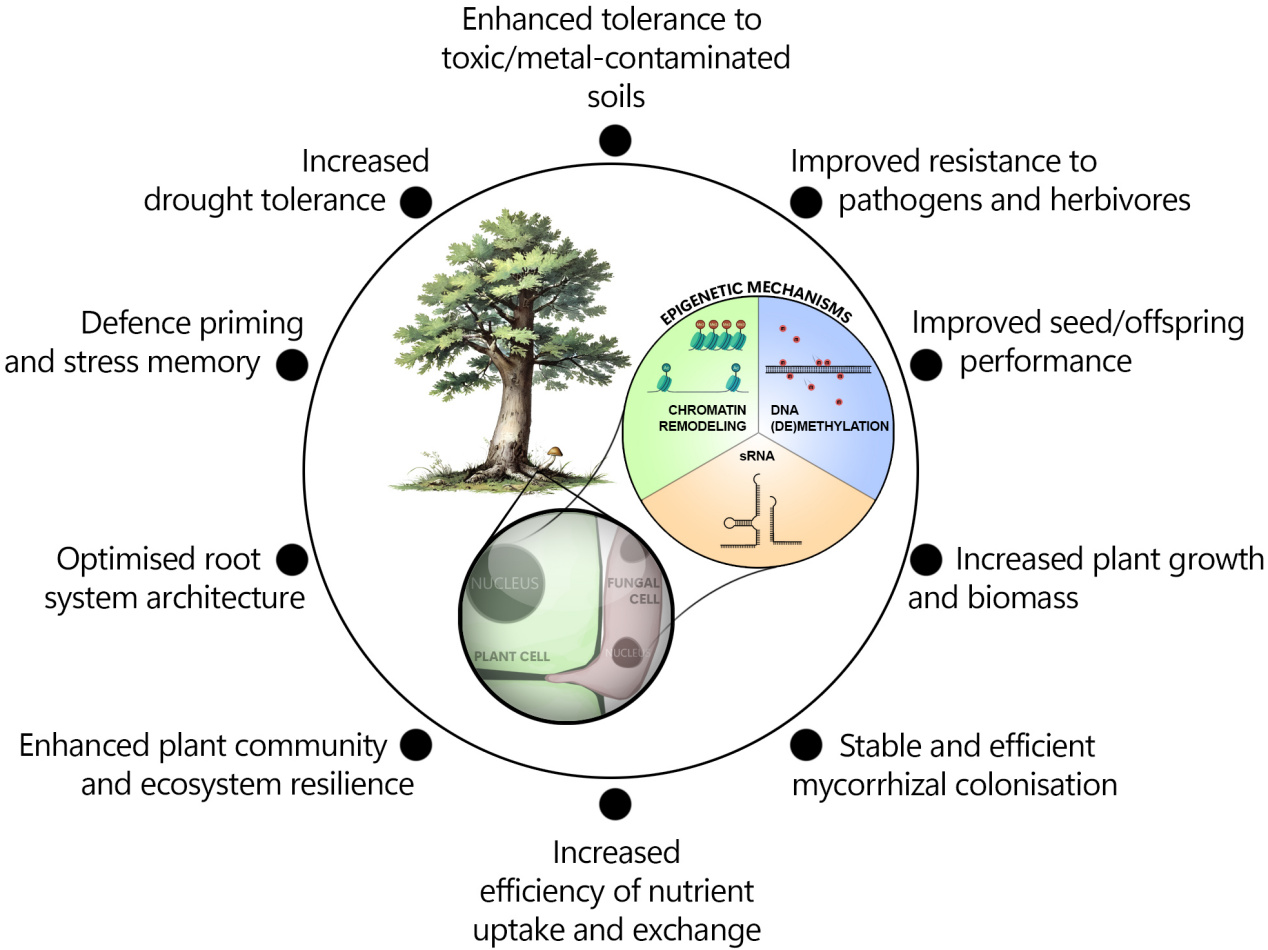
Epigenetic events occurring in plant–fungus interactions and their consequences.

## Glossary

ABA: abscisic acid
AGO: Argonaute
AM: arbuscular mycorrhiza
AMF: arbuscular mycorrhizal fungi
AMS: arbuscular mycorrhizal symbiosis
CCaMK: calcium- and calmodulin-dependent protein kinase
CC-NLR: coiled-coil nucleotide-binding leucine-rich repeat
CMN: common mycorrhizal networks
DCL: Dicer-like
DDM1: Decrease in DNA Methylation 1
DML: DNA demethylase (Demeter-like)
DSE: dark septate endophytes
EcM: ectomycorrhizas
EMF: ectomycorrhizal fungi
ERF3: ethylene-responsive transcription factor 3
ErM: ericoid mycorrhiza
GRAS: GAI/RGA/SCR transcription factor family
GRF: growth-regulating factor
HAT: histone acetyltransferase
HDAC: histone deacetylase
HD-ZIP III: class III homeodomain leucine zipper
IAA: indole-3-acetic acid
JA: jasmonic acid
LOX: lipoxygenase
MiSSP: mycorrhiza-induced small secreted protein
MSAP: Methylation-Sensitive Amplified Polymorphism
NBS-LRR: Nucleotide-binding site–Leucine-rich repeat
NSP2: Nodulation Signaling Pathway 2
OM: orchid mycorrhiza
PAL: phenylalanine ammonia-lyase
PPA: pre-penetration apparatus
PPO: polyphenol oxidase
PPR: Pentatricopeptide repeat
PR: pathogenesis-related
SA: salicylic acid
SAR: systemic acquired resistance
SSP: small secreted protein
TE: transposable element
VOC: volatile organic compound
